# The Effect of Enterohemorrhagic *E. coli* Infection on the Cell Mechanics of Host Cells

**DOI:** 10.1371/journal.pone.0112137

**Published:** 2014-11-04

**Authors:** Yin-Quan Chen, Pin-Tzu Su, Yu-Hsuan Chen, Ming-Tzo Wei, Chien-Hsiu Huang, Kathryn Osterday, Juan C. del Álamo, Wan-Jr Syu, Arthur Chiou

**Affiliations:** 1 Institute of Biophotonics, National Yang-Ming University, Taipei, Taiwan, Republic of China; 2 Institute of Microbiology & Immunology, National Yang-Ming University, Taipei, Taiwan, Republic of China; 3 Biophotonics & Molecular Imaging Research Center (BMIRC), National Yang-Ming University, Taipei, Taiwan, Republic of China; 4 Bioengineering Program, Lehigh University, Bethlehem, PA, United States of America; 5 Department of Mechanical and Aerospace Engineering, San Diego, California, United States of America; 6 Institute of Engineering in Medicine, University of California San Diego, San Diego, California, United States of America; University of Muenster, Germany

## Abstract

Enterohaemorrhagic *E. coli* (EHEC) is a type of human pathogenic bacteria. The main virulence characteristics of EHEC include the formation of attaching and effacing lesions (A/E lesions) and the production of one or more Shiga-like toxins, which may induce human uremic complications. When EHEC infects host cells, it releases translocated intimin receptor (Tir) and effector proteins inside the host cells, inducing the rearrangement and accumulation of the F-actin cytoskeleton, a phenotype leading to the formation of pedestals in the apical cell surface, and the growth of stress fibers at the base of the cells. To examine the effect of EHEC infection on cell mechanics, we carried out a series of experiments to examine HeLa cells with and without EHEC infection to quantify the changes in (1) focal adhesion area, visualized by anti-vinculin staining; (2) the distribution and orientation of stress fibers; and (3) the intracellular viscoelasticity, via directional video particle tracking microrheology. Our results indicated that in EHEC-infected HeLa cells, the focal adhesion area increased and the actin stress fibers became thicker and more aligned. The cytoskeletal reorganization induced by EHEC infection mediated a dramatic increase in the cytoplasmic elastic shear modulus of the infected cells, and a transition in the viscoelastic behavior of the cells from viscous-like to elastic-like. These changes in mechanobiological characteristics might modulate the attachments between EHEC and the host cell to withstand exfoliation, and between the host cell and the extracellular matrix, and might also alter epithelial integrity.

## Introduction

The dynamic organization of the actin cytoskeleton plays a critical role in regulating cell mechanics, including focal adhesions, rheology, and motility [1]. These aspects are related to various physiological and pathological functions such as cell division [2], proliferation [3], differentiation [4], [5], invasion [6] and metastasis [7], [8]. The dynamics of cytoskeletal organization include nucleation, polymerization and depolymerization, branching, cross-linking, and bundling actin filaments into actin stress fibers [1]. The effects of chemical and physical stimuli on cytoskeletal organization and cell mechanics have been widely reported in the literature [4], [9]–[17]. The dynamic changes in the actin cytoskeleton also play an important role in pathogen-host interactions [18], [19]. Many bacterial pathogens induce actin polymerization for efficient infection of host cells [20].

Enterohaemorrhagic *E. coli* (EHEC) is a bacterium that was first separated from contaminated hamburgers in 1982 [21]. The symptoms of the diseases caused by EHEC include abdominal cramps, diarrhea, and haemorrhagic colitis. Histopathological studies indicate that EHEC colonizes the large intestinal mucosa and induces attaching and effacing (A/E) lesions, which are characterized by the destruction of intestinal microvilli and the formation of a polymerized actin structure (known as pedestal) immediately underneath the bacterium [22], [23]. The genetic element attributed to this bacterial phenotype (or the pathogenic island) is also known as the locus of enterocyte effacement island; it contains all genes to encode a type III secretion system, which injects effector proteins into host cells to harass the host cells functioning for the bacterial benefit. As the translocated intimin receptor (Tir), one of the effector proteins, reaches the host cell membrane, it forms a binding site for the bacterial outer membrane protein intimin [24]. The Tir-intimin interaction triggers signals for actin polymerization and results in the formation of the pedestal [23], [25]. Previous studies indicate that Tir is the main factor to induce actin polymerization in the host cells during EHEC infection. Actin polymerization, rearrangement, and accumulation are also induced when Tir alone is expressed directly in cells by transfection [24], [26]. These changes in actin organization are expected to affect intracellular complex shear modulus, which may affect the epithelial function by modulating the transmission of mechanical forces within the cell [27]. However, the changes in mechanical properties of EHEC-infected cells are not well studied. In this paper, we used confocal fluorescence microscopy and directional video particle tracking microrheology (DVPTM) to study the effects of EHEC infection on the actin cytoskeleton, focal adhesions and intracellular viscoelasticity of the host cell.

Video particle tracking microrheology (VPTM) is a technique to measure the local shear moduli (both viscous and elastic) of complex materials with a spatial resolution on the order of a few microns, and requiring sample volumes on the order of only a few micro-liters [2], [8], [28]–[36]. A typical experimental setup includes a microscope stage equipped with an objective lens and a charge-coupled device (CCD) camera linked to a computer to record the motion of micron-size particles in the test sample. Tracking and analysis of the particle motion allows researchers to quantify the sample's mechanical properties. In directional video particle tracking microrheology (DVPTM), additional analyses are performed to compute the shear moduli along different directions [37]–[39]. This is important because cells often re-align their cytoskeleton in response to external stimuli leading to marked differences in their intracellular viscoelastic properties along different directions [37], [40]–[42].

Our experiments revealed that EHEC induces actin rearrangement to form stress fibers that are thicker and more aligned in the basal region of the host cell. EHEC infection also led to increased focal adhesion area. This cytoskeletal reorganization caused important changes in the mechanical properties of the host cell, including a marked increase in the elastic shear modulus, a transition from a viscous-like behavior to an elastic-like behavior, and a directional polarization of the viscoelastic properties. These mechanical changes could vary the adherence between EHEC and the host cell or between the host cell and extracellular matrix.

## Materials and Methods

### Bacterial strains

Wild-type enterohemorrhagic Escherichia *E. coli* O157:H7 strains (ATCC 43888) were used in this study. Bacteria were cultured in LB (Luria-Bertani; 244620, Difco) media at 37°C with continuous agitation. Ampicillin (100 µg/mL), kanamycin (50 µg/mL) and chloramphenicol (20 µg/mL) were appropriately incorporated into the media when plasmid-transformed bacteria were used.

### Cell culture

HeLa cells (ATCC CCL2) were grown in Dulbecco's modified Eagle medium (DMEM) supplemented with 10% fetal bovine serum (Gibco) and 1× antibiotic–antimycotic (15240-062, Gibco) at 37°C under 5% CO_2_.

### Infection assay and immunofluorescence staining

HeLa cells (2×10^5^ cells) were seeded overnight on 18-mm glass coverslips (Dechglaser) in six-well tissue culture plates. The cells were washed with phosphate-buffered saline (PBS) and the wells were replenished with DMEM (Gibco). Prior to the infection assay, bacteria were grown on LB agar plates and a single colony was picked up for inoculation in LB broth. The culture was incubated overnight at 37°C, and the overnight cultured bacteria were 1∶50 diluted; the solution containing approximately 5×10^6^ bacteria was added to ∼2×10^5^ cells (i.e., ∼25 bacteria/cell) to allow bacterial infection for 6 hours at 37°C in the presence of 5% CO_2_. Thereafter, bacteria not adhered to the cells were removed by washing off with DMEM.

The infected HeLa cells were washed with PBS (at room temperature) twice and fixed with 4% formaldehyde (Sigma) in the same buffer for 30 minutes at 37°C. The cells were then permeated with 0.5% Triton X-100 (45-000-229, Plusone) at room temperature for 15 minutes and blocked with 3% bovine serum albumin in PBS at 4°C overnight. To observe the EHEC bacteria, rabbit anti-O157 antibody (Difco) was used. The bound primary antibody was detected by Alexa Fluor 405-conjugated goat anti-rabbit IgG (A-31556, Life Technologies). Cellular actin was stained with FITC-labeled phalloidin (P5282, Sigma) for 20 minutes at room temperature. To detect vinculin by indirect immunofluorescence, cells were fixed and stained with mouse-anti-vinculin antibody (V9131, Sigma), followed by staining with goat-anti-mouse-TRITC. The stained cells were examined via a Zeiss LSM 700 laser confocal microscope with a 100X/1.40 N.A. oil immersion objective.

### Fluorescence live imaging of HeLa cells under EHEC infection

HeLa cells (2×10^5^ cells), expressing the enhanced yellow fluorescent protein-actin (fluorescence excitation/admission peaks: 513 nm/526 nm; encoded by pEYFP-Actin), were seeded on the 3.5-cm glass-bottom culture dishes (MatTek) coated with poly-L-lysine. Cells were grown overnight in Dulbecco's modified Eagle medium (DMEM) with 10% fetal bovine serum at 37°C in 5% CO_2_. The cells were then washed with PBS and the media was replaced with phenol red-free DMEM (GibcoBRL) containing ampicillin (100 µg/mL). To these cells, overnight-cultured bacteria harboring pQE60-RFP, which encodes red fluorescent protein (fluorescence excitation/emission peaks: 584 nm/607 nm), were added at a dilution of 1∶50 and left for 6 hours at 37°C and 5% CO_2_ in an incubator. After washing with PBS, the dishes were replenished again with red-free DMEM-containing 100 µg/mL ampicillin. The dish was then moved to an incubator chamber (Chamlide TC, Live Cell Instrument) (maintained at 37°C in 5% CO_2_) mounted on a Nikon (Eclipse Ti) inverted microscope equipped with a 100X/N.A 1.45 oil objective.

### Video particle tracking microrheology

HeLa cells (2×10^5^ cells/mL) were infected with EHEC as described above in 3.5-cm glass-bottom culture dishes (αPLUS). Cells with and without (i.e. control) EHEC infection were ballistically injected with 20 µL carboxylated polystyrene particles (F8807, Invitrogen, fluorescence excitation/admission peaks: 660 nm/680 nm, diameter = 200 nm, concentration: 1.35×10^12^ particles/mL) via a biolistic particle delivery system (PDS-100, Bio-Rad; pressure 450 psi). Cells were then washed with PBS twice and incubated for 1 hour in 2 mL DMEM.

To track and record the motion of the intracellular fluorescent particles, an inverted epifluorescence microscope (Nikon Eclipse Ti) equipped with an oil-immersion objective (Nikon, 100X/N.A. 1.45) and a CCD camera (Hamamatsu, OHCA-Flash 4.0) were used. Time-lapse image sequences were recorded for 10 sec at a frame rate of 100 Hz. These sequences were long enough to obtain converged statistics (1000 time points) while at the same time avoiding potential phototoxic effects, particularly damage to the F-actin cytoskeleton induced by the fluorescent beads [43].

We analyzed the two-dimensional motion of the embedded particles in the apical and the basal regions of the HeLa (harboring pEYFP-Actin) cells without and with EHEC infection. From the trajectory [x(t) and y(t), as a function of time (t)] of each particle, we calculated the ensemble-averaged mean squared displacement (MSD), <Δr^2^(τ)> = <[*x*(*t+τ*)−*x*(*t*)]^2^+[*y*(*t+τ*)−*y*(*t*)]^2^>, and determined the principal directions along which MSD is maximal and minimal. The intracellular viscoelastic properties (elastic shear modulus G′; viscous shear modulus G″) were deduced along the principal directions of the MSD (for α<1) through a pseudo-Stokes Einstein relation that was recently derived for nematic media [39]. In order to ensure that the data used to estimate G′ and G″ was consistent with the hypothesis of thermodynamical equilibrium, we discarded trajectories of particles undergoing directional transport (35 out of 391; ∼9%), and limited our analysis to the range of time lags 0.01 s<τ<0.1 s. Particles undergoing directional transport were detected by fitting the mean square displacement (MSD = <Δr^2^(τ)>) to the power law τ^α^ with exponent (α), and finding those particles with α>1.

### Statistical Analyses

Statistical analysis of angular variables including calculation of variance and kurtosis was performed using specific procedures for circular distributions [44]. We tested if the measured samples of stress-fiber orientation without and with EHEC infection came from similar circular distributions using Watson's U_2_ non-parametric test. The same test was used to compare the principal directions of MSD without and with EHEC infection. Statistical analysis of non-circular variables such as focal adhesion number, focal adhesion area or intracellular shear modulus was performed using standard procedures. Samples from different cases were compared using a Student's *t* test.

## Results

### EHEC Infection Increases Focal Adhesion Area in HeLa Cells

**Figure 1:**
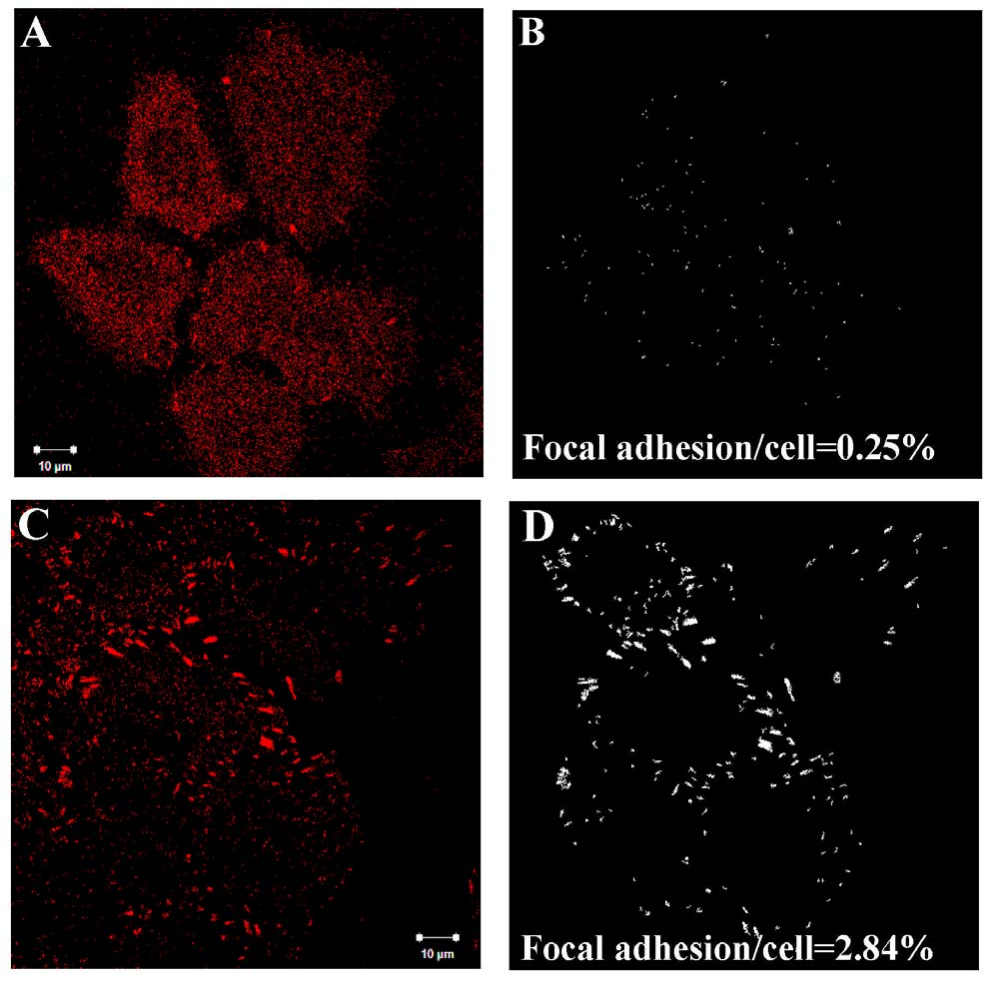
Focal adhesion in HeLa cells without *vs.* with EHEC infection. Representative confocal micrographs of HeLa cells (A) without- and (C) with- EHEC infection; vinculin was detected by indirect immunofluorescence. The images of focal adhesion were segmented by setting an intensity threshold via MetaMorph software to calculate the fractional area of focal adhesion, as shown in (B) for HeLa cells without-, and (D) with- EHEC infection.

**Figure 2:**
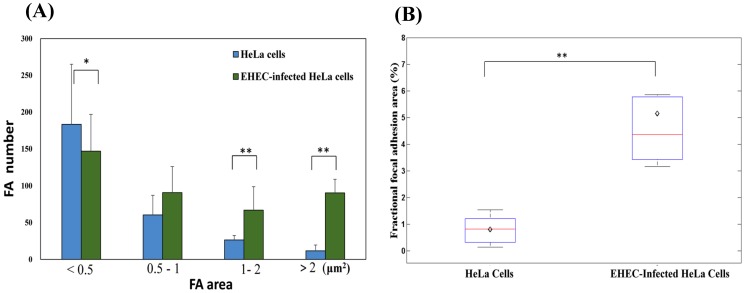
Focal adhesion number and size in HeLa cells without *vs.* with EHEC infection. (A) Number of focal adhesions as a function of their area. The mean values and standard deviations are indicated by the height of the thick bars and the thin lines, respectively. (B) Fraction of cell area occupied by focal adhesions. The symbol “⋄” and the horizontal bar inside each box represent the mean and the median values, respectively; and the lower and the upper sides of each box represent the values at the first and the third quartiles, respectively. *: *p*<0.05; **: *p*<0.001; data obtained from N = 15 cells.

The effect of EHEC infection on the regulation of focal adhesions (FA) in the host cells was visualized by anti-vinculin staining. Fig. 1A shows cells without EHEC infection and Fig. 1C shows cells that had been infected for 6 hours. Analysis of the vinculin area in both cases revealed that EHEC infection induced an increase in the number of large FA (area >1 µm^2^), and a corresponding reduction in the number of small FA (area <0.5 µm^2^) (Fig. 2A). Additionally, the fraction of cell area occupied by focal adhesions (defined as the ratio of the total focal adhesion area in each cell to the total area enclosed by the boundary of each cell) increased from 0.79% to 5.14% after EHEC infection (Fig. 2B).

### EHEC Infection Induces Actin Rearrangement and Accumulation

**Figure 3:**
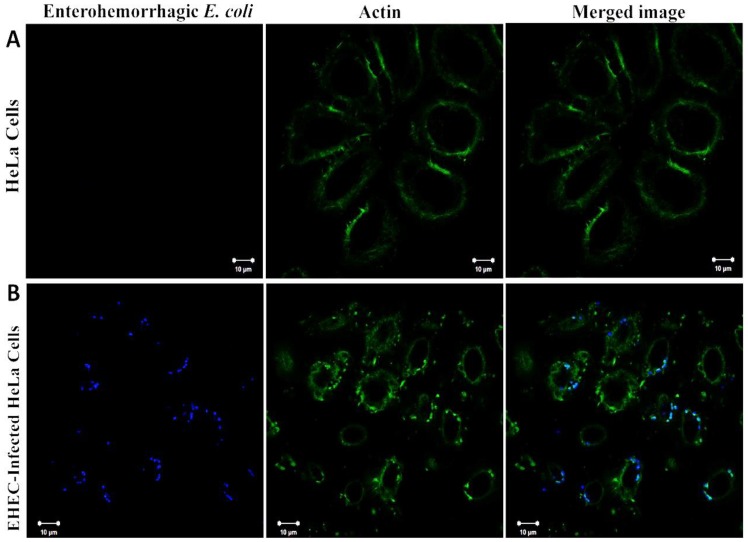
Immunofluorescence microscopy images of the apical region of HeLa cells without *vs.* with EHEC infection. (A) HeLa cells without EHEC infection; (B) HeLa cells, after being infected with EHEC for 6 hours. HeLa cells were fixed, permeated and stained: green, FITC-label phalloidin for actin; blue, rabbit anti-O157 and Alexa Fluor 405-goat anti-rabbit IgG for EHEC. The merged images in turquoise represent the EHEC induced pedestal formation.

**Figure 4:**
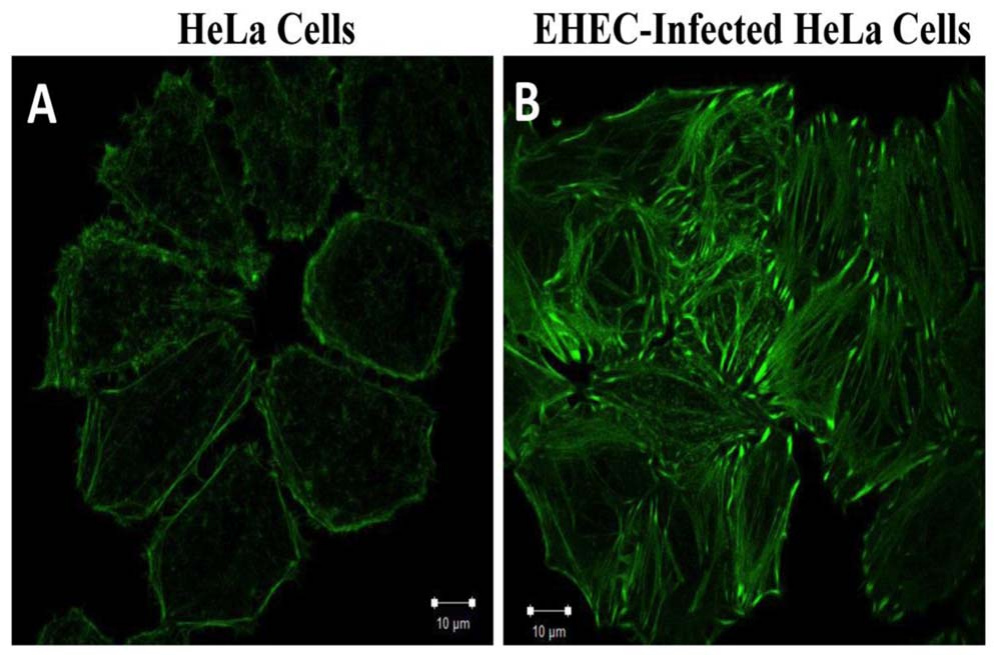
Confocal micrographs of actin filaments, FITC-labeled phalloidin (green), in the basal region of (A) HeLa cells without EHEC infection; (B) HeLa cells after infected by EHEC for 6 hours. The formation of the stress fibers in the basal region of HeLa cells after EHEC infection can be clearly observed in (B).

We labeled EHEC by Alexa Fluor 405 (blue) and actin via FITC-phalloidin staining (green) and examined the apical and basal regions of HeLa cells without EHEC infection vs. cells that had been infected for 6 hours. The resulting images revealed actin accumulation consistent with the formation of pedestals in the apical region of the infected HeLa cells (Fig. 3B). As expected, the pedestals were not observed in the control HeLa cells (Fig. 3A). Besides, EHEC infection induced actin polymerization and bundling into stress fibers in the basal region of the cells (Fig. 4B), but the stress fibers were less discernable in the basal region of the non-infected cells (Fig. 4A).

**Figure 5:**
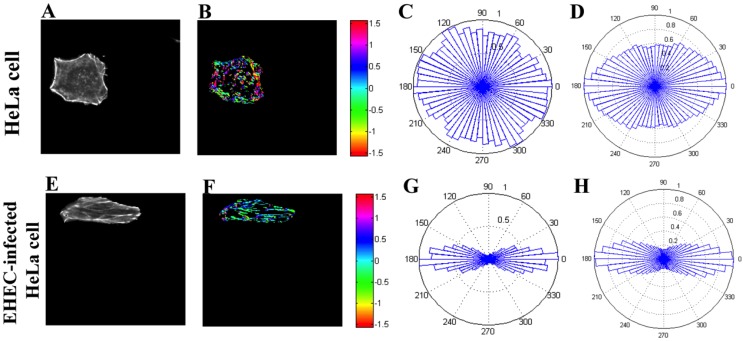
The F-actin orientation in the basal region of HeLa cells (A to D) without, and (E to H) with EHEC infection. Cells fixed and stained with FITC-phalloidin (green) for F-actin, imaged in HeLa cells (A) without, (E) with EHEC infection. In (B) and (F), the images of actin fibers with fluorescence intensity above a selected threshold are marked and color-coded to represent their angular orientation in radians (1 radian≈57.3 degrees). The direction along the principal axis (i.e., the direction along which the maximum number of actin filaments were aligned) is denoted as 0; (B) without, and (F) with EHEC infection. The angular plots corresponding to (B) and (F) are shown respectively in panels (C) and (G), in degrees. Similar data compiled from from 15 cells are shown in (D) and (H), respectively. The orientation distributions without and with EHEC infection are statistically significant (p<0.001, Watson's U_2_ test). Variance and Kurtosis of the angular distributions are given in Table 1.

Our analysis of the orientation of the stress fibers in the basal region of HeLa cells indicated that these fibers became better aligned in EHEC-infected cells than in non-infected cells, as shown in Fig. 5. Panels (A) and (E) in this figure are the original fluorescence images of F-actin for two example cells without and with EHEC infection, respectively. These images are thresholded to create binary images containing only the fibers, which are color-coded to represent the orientations of the fibers in panels (B) and (D), and the distributions of fiber orientation angles are shown in panels (C) and (G). The direction along the principal axis (i.e., the preferred direction of the actin filaments for each cell) is denoted as 0 (180) degrees. The results suggest that the angular distribution is narrower after EHEC infection.

To demonstrate that EHEC infection of HeLa cells led to stress-fiber alignment, we compiled angular distributions of stress fibers for 15 cells with and without infection (Fig. 5D and Fig. 5H, respectively). Statistical analysis of stress-fiber orientation revealed that the variance of the angular distribution decreased and its kurtosis increased with EHEC infection (see Table 1). These differences were found to be statistically significant (p<0.001, Watson's U_2_ test). Because the variance and kurtosis of a distribution respectively quantify its spread and peakedness, this analysis confirms that the orientation of the F-actin cytoskeleton became tightly aligned along a common direction after EHEC infection. These observed directional changes in the cell cytoskeleton suggest that EHEC infection may induce anisotropy in intracellular rheology, meaning that the viscoelastic properties of the infected cells could be different when measured along different directions. This led to a subsequent study, via DVPTM, of the viscoelastic properties of HeLa cells with vs. without EHEC infection.

**Table 1:** Variance and Kurtosis of the angular distribution of stress fiber orientation shown in Fig. 6.

	Angular Variance (deg^2^)	Kurtosis
**Normal HeLa cells**	1020	0.12
**EHEC-infected HeLa cells**	590	0.45

EHEC infection leads to tight stress fiber alignment thus yielding lower variance and higher Kurtosis.

### EHEC-Infection Leads to Anisotropic Viscoelastic Properties in HeLa Cells

We applied DVPTM to analyze the effect of EHEC infection on the intracellular shear modulus of HeLa cells with and without EHEC infection. To assess the changes in shear modulus induced by both pedestal formation and stress-fiber reinforcement and alignment, we tracked the motion of embedded particles on two different z-planes, one located in the apical and the other in the basal regions of each cell. From the tracked particle trajectories, we calculated the mean squared displacements (MSDs) as a function of time lag, τ. Because EHEC infection caused a marked realignment of stress fibers in the basal region of the cell, we analyzed the MSDs along different directions.

To assess whether the results are consistent with the theoretical results for ideal Brownian motion, and to quantify the viscoelastic nature of their cytoplasm, the MSDs were fit to the power law <Δr^2^(τ)> = Aτ^α^. The exponent α characterizes the diffusive behavior of the probing particles; α>1 indicates active transport; α = 1 indicates ideal Brownian diffusion in a Newtonian viscous fluid, and α = 0 reflects trapping in purely elastic media [28], [45]. Hence, in general, the exponent α reflects whether the material is more elastic-like or more viscous-like. On the other hand, the proportionality constant “A” indicates the level of viscoelastic resistance encountered by the probing particles in their motion; high polymer density and stable cross-linking lead to stronger resistance to particle motion and the extent of MSD becomes smaller. Active random-like fluctuations of the F-actin network caused by motor proteins may add a contribution to the MSDs with α≈1 for frequencies lower than 10 Hz [46]. This active contribution would be impossible to differentiate from ideal Brownian diffusion and could lead us to underestimate intracellular viscoelasticity. Thus, to ensure that our results are not contaminated by this potential effect, only the experimental data within the time lag range of 0.01 s≤τ≤0.1 s were taken into account in our analysis.

**Figure 6:**
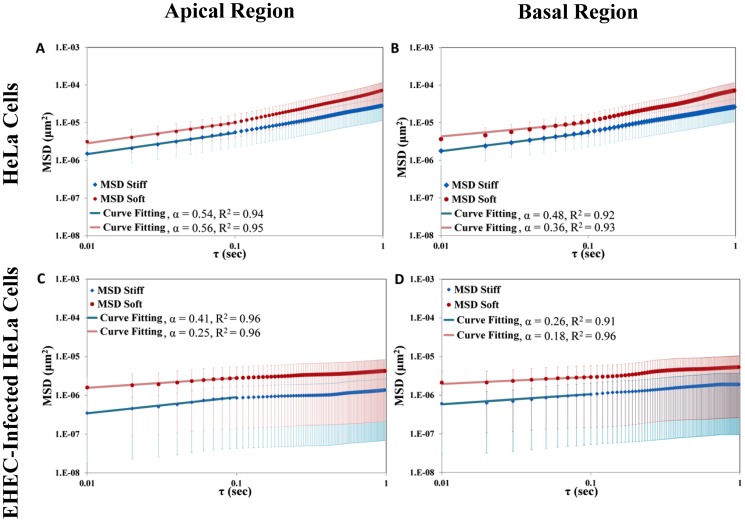
The principal value of the mean squared displacement (PMSD) as a function of the time lag (τ). In each diagram, the upper curve represents the result for the soft axis, and the lower curve for the stiff axis. (A) Apical region (deduced from 112 particles) of HeLa cells without EHEC infection; (B) basal region of HeLa cells without EHEC infection (deduced from 158 particles); (C) apical region of EHEC-infected HeLa cells (deduced from 66 particles); (D) basal region of EHEC-infected HeLa cells (deduced from 55 particles). The straight lines represent the power law dependence; the symbols represent the mean value of the experimental results. The vertical bars around each point represent the corresponding standard deviation.

Fig. 6 shows the measured MSD for particles in the apical and the basal regions of HeLa cells, without vs. with EHEC infection. In two-dimensional motion, the MSD of a particle is a 2×2 symmetric matrix with two perpendicular principal directions. If the medium is isotropic, then the principal values (eigenvalues) of the MSD matrix are equal. However, if the medium is anisotropic the principal values will differ, revealing the two perpendicular directions in which the medium is softest (i.e. soft direction, maximal MSD) and stiffest (i.e. stiff direction, minimal MSD) [37], [47]. Thus, to test the hypothesis that the structural changes in the cell cytoskeleton after EHEC infection induced anisotropy in intracellular rheology, we compared the principal values of the MSD. In the control case, the mean values of MSD along the soft direction are about 2 times the corresponding values along the stiff direction (Fig. 6: A, B). In the case of EHEC-infected cells, the mean values of MSD along the soft direction are about 5 to 6 times the corresponding values along the stiff direction (Fig. 6: C, D). These results indicate that the degree of anisotropy in MSD increases with EHEC infection. The dependence of the MSD on the time lag τ also showed significant differences between the control vs. the EHEC-infected samples. The power law exponent α was measured to be in the range of 0.36 to 0.56 in both the apical and the basal regions of HeLa cells without EHEC infection. In contrast, the value of “α” decreased to a lower value in the range of 0.18 to 0.41, indicating that, after EHEC infection, the cytoplasmic microenvironment became more elastic-like than viscous-like. Interestingly, Fig. 6 also reveals that the variations in the distribution of MSD for different probing particles (see error bars in the plots) are much higher for infected cells than for controls, suggesting that EHEC infection may cause a more heterogeneous spatial distribution of viscoelasticity in the host cells.

**Figure 7:**
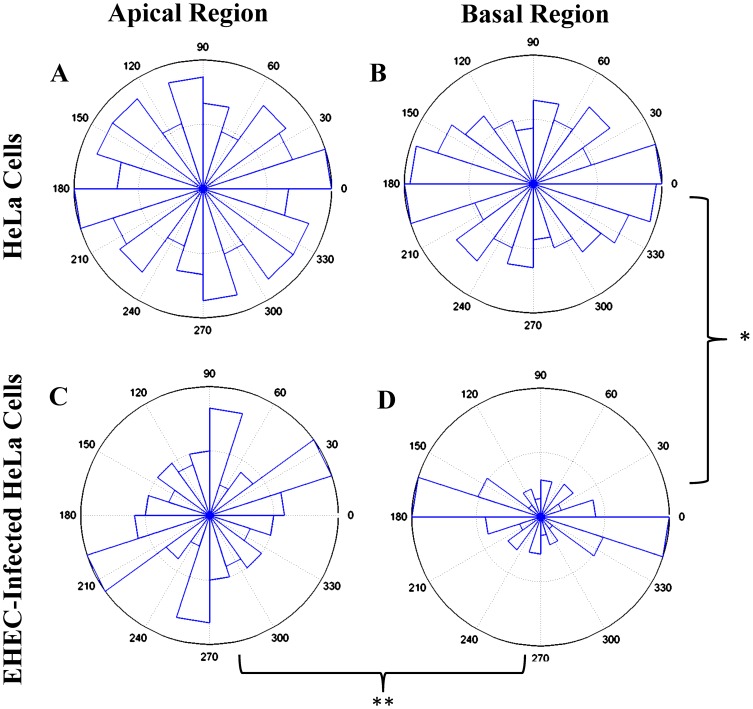
Angular distribution of the direction of maximum mean squared displacement (soft direction), *θ_MSD,max_*, in the apical and the basal regions of HeLa cells without *vs.* with EHEC infection. (A) Apical region of HeLa cells without EHEC infection (data deduced from 112 particles; (B) basal region of HeLa cells without EHEC infection (data deduced from 158 particles); (C) apical region of EHEC-infected HeLa cells (data deduced from 66 particles); (D) basal region of EHEC-infected HeLa cells (data deduced from 55 particles). Statistically significant differences (obtained from Watson's U_2_ test for angular distributions) were found between apical and basal regions of EHEC-infected cells (**, p<0.01), and between basal regions of non-infected and EHEC-infected cells (*, p<0.05). Variance and kurtosis of the angular distributions of *θ_MSD,max_* are given in Table 2.

Fig. 7 and Table 2 summarize the distribution of angular directions of maximum MSD (*θ_MSD,max_*) for τ = 0.1 s. In cells without EHEC infection, the overall distribution of *θ_MSD,max_* was fairly uniform, yielding high angular variances and low kurtosis coefficients, especially in the apical cell region. Similarly, the apical region of EHEC-infected cells had no significant polarization in the distribution of *θ_MSD,max_*. However, the basal region of the infected cells had highly polarized rheological properties (Fig. 7C) in consistence with the well-aligned orientation of their stress fibers. The angular distribution of *θ_MSD,max_* had the smallest variance and highest kurtosis in this case and these differences were statistically significant (Table 2).

**Table 2:** Variance and Kurtosis of the angular distribution of the direction of normalized maximum MSD, *θ_MSD,max,_* at the apical and the basal regions of HeLa cells with and without EHEC infection.

		Angular Variance (deg^2^)	Kurtosis
**Normal HeLa cells**	**Basal region**	1020	0.15
	**Apical region**	1080	0.09
**EHEC-infected HeLa cells**	**Basal region**	790	0.35
	**Apical region**	1210	0.03

EHEC infection leads to anisotropic intracellular rheology at the basal region of the cells, causing a lower variance and higher Kurtosis of the angular distribution of *θ_MSD,max_*.

### EHEC-Infection Induces Intracellular Hardening in HeLa Cells

Our observation that EHEC infection induced actin polymerization in the apical region of HeLa cells and stress-fiber reinforcement in their basal region led us to measure the intracellular viscoelastic properties of these cells without vs. with EHEC infection. The intracellular elastic shear modulus, G′, and viscous shear modulus, G″, of HeLa cells were deduced from the MSD using recently derived expressions for the viscoelastic resistance experienced by a particle moving inside a nematic fluid [39].

**Figure 8:**
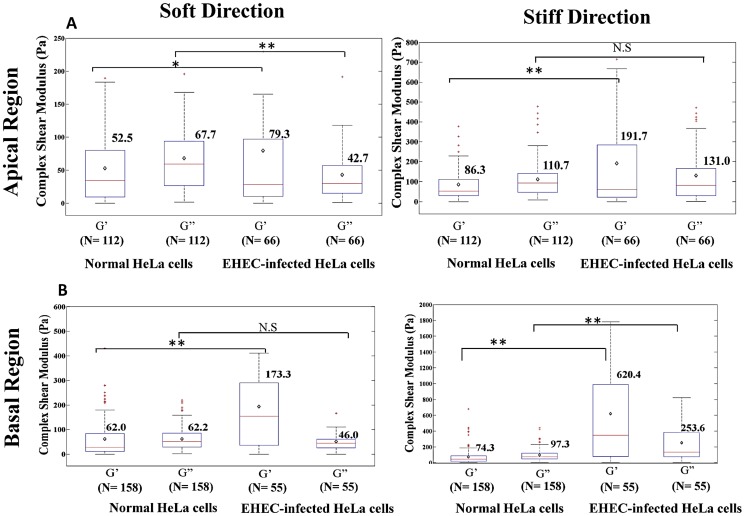
Boxplot of intracellular elastic shear modulus G′ and viscous shear modulus G″ (at 10 Hz) of HeLa cells without vs. with EHEC infection. Left: along the soft axis, and Right: along the stiff axis; (A) the apical region, and (B) the basal region. *: *p*<0.05; **: *p*<0.001. The symbol “⋄” and the horizontal bar inside each box represent the mean and the median values, respectively; and the lower and the upper sides of each box represent the values at the first and the third quartiles, respectively.

Our results for the elastic (G′) and viscous (G″) shear moduli at a frequency f = 10 Hz are compared in Fig. 8, and their mean values are summarized in Tables 3, 4, and 5, along with the standard error of the mean (SEM). A first look at the data indicates that after EHEC infection the elastic shear modulus G′ increases along both the stiff and the soft axes, and both in the apical and the basal regions of the cell. However, the most significant changes were observed for the elastic shear modulus, G′, while the viscous shear modulus, G″, experienced a modest increase. Consequently, EHEC infection caused a transition in the viscoelastic nature of the cells from viscous-like behavior (G″>G′) to elastic-like behavior (G′>G″). In the apical region of the cells, G′ increased in the stiff direction by over a factor of 2 due to the formation of actin pedestals, while it experienced a modest increase in the soft direction. Consistent with the changes in actin polymerization and reorganization observed Fig. 4B, EHEC infection caused a dramatic increase of the elastic shear modulus in the basal region of the cells; G′ increased by a factor of ∼8 along the stiff direction and by a factor of ∼3 along the soft direction. In addition to these changes, the ratios G′_stiff_/G′_soft_ and G″_stiff_/G″_soft_ grew significantly with EHEC infection, especially in the basal region of the cell (Table 5), indicating that the viscoelastic properties of the cell became more anisotropic. This result is in good agreement with the alignment of the stress fibers observed by fluorescence imaging (Fig. 5).

**Table 3:** The mean and the standard error of the mean (SEM) of the elastic shear modulus (G′) and viscous shear modulus (G″) at 10 Hz in the apical region of HeLa cells without vs. with EHEC infection.

Apical Region	Soft Direction		Stiff Direction	
Complex shear modulus (Pa)	G′	G″	G′	G″
**Normal HeLa cells**	52.5±5.6	67.7±5.6	86.3±11.8	110.7±8.6
**EHEC-infected HeLa cells**	79.3±11.4	42.7±4.6	191.7±28.8	131.0±15.6
**Relative change**	**51%**	**−37%**	**122%**	**N.S**

**Table 4:** The mean and the standard error of the mean (SEM) of the elastic shear modulus (G′) and viscous shear modulus (G″) at 10 Hz in the basal region of HeLa cells without vs. with EHEC infection.

Basal Region	Soft Direction		Stiff Direction	
Complex shear modulus (Pa)	G′	G″	G′	G″
**Normal HeLa cells**	62.0±6.0	62.2±3.8	74.3±7.8	97.3±6.4
**EHEC-infected HeLa cells**	173.3±21.1	46.0±4.5	620.4±72.7	253.6±25.8
**Relative change**	**180%**	**N.S**	**735%**	**161%**

**Table 5:** The ratio of viscoelastic shear moduli in the stiff direction and the corresponding values in the soft direction for HeLa cells without vs. with EHEC infection.

	Apical Regions		Basal Regions	
Ratio of Stiff and Soft	G′_stiff/soft_	G″_stiff/soft_	G′_stiff/soft_	G″_stiff/soft_
**Normal HeLa cells**	**1.6**	**1.6**	1.2	1.6
**EHEC-infected HeLa cells**	**2.4**	**3.1**	3.6	5.5

## Discussion

Epithelial and endothelial permeability depends on a delicate balance of mechanical forces that is regulated by the cell cytoskeleton, which, in turn, is modulated by mechanical forces [14], [27], [48]. The transmission of these forces throughout the cell depends on the strength of focal adhesions, and the directionality and magnitude of intracellular viscoelastic properties. Enteric pathogens of EPEC and EHEC infect the intestinal tract to cause attaching and effacing lesions, a phenomenon that is due to rearrangement of actin filaments in the infected cells. In 1996, Rosenshine et al. first found that EPEC could trigger epithelial signals in HeLa cells to mediate actin rearrangement [49]. In 1999, DeVinney et al. also reported that EHEC infects HeLa cells causing similar actin rearrangements [50]. In this paper, we report the effect of EHEC infection on the cell mechanics of HeLa cells, including the orientation of stress fibers, focal adhesion area, and intracellular viscoelastic properties. We note that EHEC is similar to EPEC (enteropathogenic *E. coli*) in type III secretion and, by analogy, may also destruct microtubule networks in host cells [51] and that may have additional effects on intracellular rheology. In this work, only the effects on actin polymerization and aggregation and the associated changes in cell mechanics were studied.

In our experiments, the elastic and viscous shear moduli of non-infected and infected HeLa cells were measured in the frequency range of 0.1 to 100 Hz by directional particle tracking microrheology (Tables 3 and 4). Our measured values for non-infected cells were comparable with those reported earlier for 3T3 fibroblasts (viscous shear modulus G″ = 30±4 Pa, and elastic shear modulus G′ = 60±5 Pa) [52]. After EHEC infection, the elastic shear modulus of the cells increased considerably while the viscous shear modulus did not increase nearly as much. Consequently, the mechanical properties of the HeLa cells transitioned from viscous-like (i.e., G″>G′) to elastic-like (G′>G″). The changes in elastic shear modulus were highest in the basal region of the cells even if EHEC bacteria adhered to the apical surface of the HeLa cells. This result is consistent with our observation of stress-fiber thickening at the bottom of the cells after EHEC infection (Fig. 4). The stress fibers also aligned along a common direction as a consequence of infection (Fig. 5), causing the mechanical properties of the cell to be more anisotropic (i.e. polarized).

Our data shows that EHEC infection led to an increase focal adhesion area and intracellular elastic shear modulus, as well as thicker and better-aligned actin stress fibers. These changes in mechanical properties could alter the adherence between EHEC and the host cell or between EHEC and extracellular matrix to resist shear flow. Previous studies have reported that in endothelial cells subjected to shear flow, increased cellular elastic shear modulus and stress fibers are associated to improved cell adhesion [53]. Generally, cellular stiffness often bears a positive correlation with cell adhesion [54], [55].
